# Case for diagnosis. Atypical Grover’s disease^☆^^☆☆^

**DOI:** 10.1016/j.abd.2020.06.011

**Published:** 2021-02-02

**Authors:** Pablo Vargas-Mora, Diego Orlandi, Irene Araya, Claudia Morales

**Affiliations:** a Department of Dermatology, Faculty of Medicine, Universidad de Chile, Santiago, Chile; b Dermopathology Section, Pathology Service, Hospital Clínico Universidad de Chile, Santiago, Chile

**Keywords:** Acantholysis, Atypical, Grover’s disease, Transient acantholytic dermatosis

## Abstract

A 55-year-old male presented with an eight-month history of erythematous papules and plaques with demarcated areas of spared skin on his trunk, upper extremities, neck, and face. Grover’s disease is a rare, acquired disorder of unknown origin, which is classically characterized by the appearance of erythematous papules on the upper trunk that are usually transient. As in the present case, there are reports of atypical disease, with facial involvement, pityriasis rubra pilaris-like lesions, and a more chronic course.

## Case report

**Figure 1:**
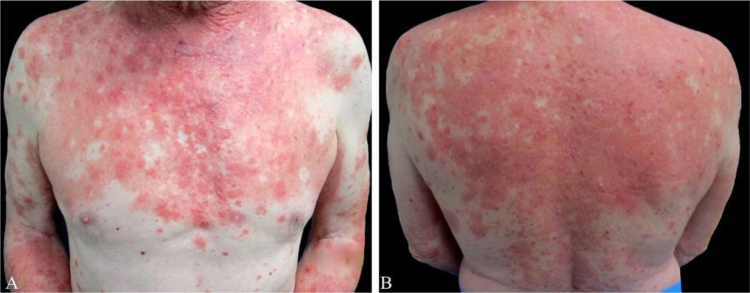
(A and B) Erythematous papules and plaques with well-demarcated areas of spared skin on anterior thorax and back.

**Figure 2:**
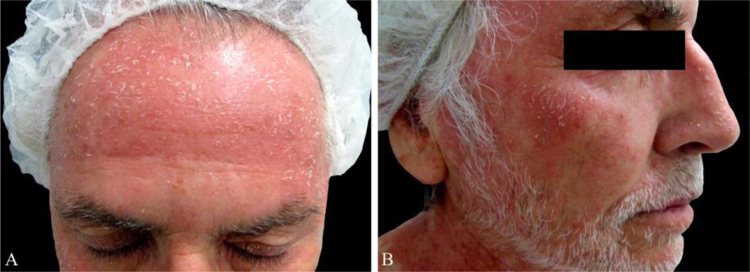
(A and B) Erythematous papules and plaques with superficial scaling on the face.

**Figure 3:**
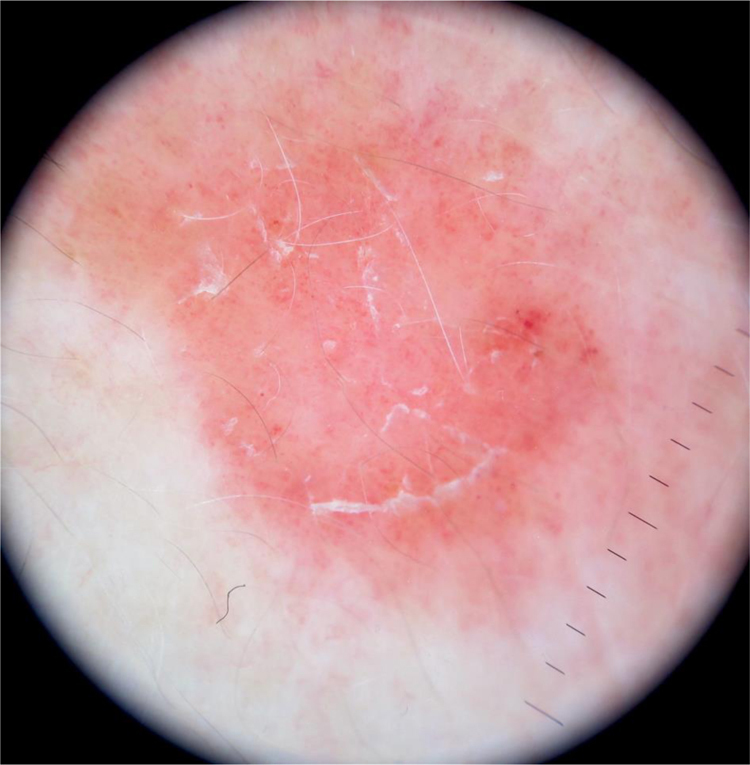
Dermoscopy shows a pink background with dotted and glomerular vessels, and fine white scaling.

A 55-year-old male presented to the dermatology clinic for the evaluation of progression of intensely pruritic erythematous papules and plaques on his trunk, upper extremities, neck, and face over eight months. On physical examination, the lesions were partially confluent with well-demarcated areas of spared skin and presented superficial fine scaling (Figure 1, Figure 2). Dermoscopy showed a homogeneous pink background with glomerular and irregular dotted vessels associated with fine superficial scaling (Fig. 3). His medical history was significant for gastroesophageal reflux disease, treated with esomeprazole.

There were no significant anomalies on general lab tests (CBC, chemistry panel, hepatic profile), and HIV, hepatitis B, hepatitis C, and VDRL serologies were all non-reactive.

**Figure 4:**
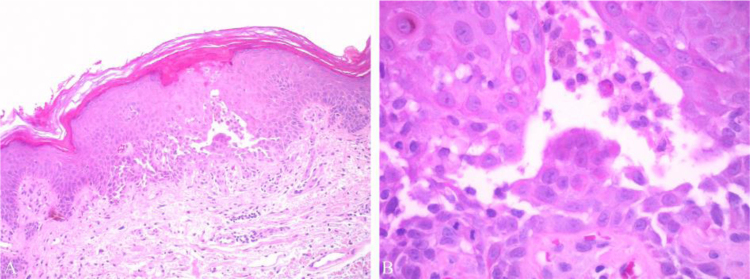
(A and B) Suprabasal acantholysis with focal spongiosis and numerous dyskeratotic cells (Hematoxylin & eosin, ×40 and ×100, respectively).

Two punch skin biopsies were taken, which showed suprabasal focal acantholysis with numerous dyskeratotic cells and eosinophils (Fig. 4).

## What is your diagnosis?

a) Pityriasis rubra pilaris
b) Atypical Grover’s disease (GD)
c) Darier’s disease
d) Pemphigus foliaceus

## Discussion

GD, also known as transient acantholytic dermatosis, is an uncommon acquired condition of unknown origin, first described by Ralph Grover in 1970.^1^, ^2^ It affects middle aged and older adults, with predominance in males (2–3:1 ratio) and Caucasians. It’s characterized by erythematous papules and occasionally vesicles, primarily on the upper trunk and proximal extremities, associated with variable pruritus.^3^, ^4^, ^5^

Although it was first described as a transient disease, lasting just for a few weeks, subsequent reports have shown that GD could last for several months or be recurrent.^1^, ^2^, ^3^

The reported case presented with extensive facial involvement, an uncommon feature of GD. Gantz et al. conducted a systematic review of 69 patients with atypical distribution GD which showed that facial or scalp lesions were present in 24% of these cases.^6^ Other atypical locations included palms, soles, axillae, inguinal folds, and dermatomeric or Blaschkoid distribution.

Another noteworthy aspect of the clinical presentation of this patient was the presence of well-demarcated areas of spared skin, which could be a strong diagnostic pitfall towards pityriasis rubra pilaris (PRP). However, there are case reports of PRP with histopathology compatible with GD and cases of GD with histopathology of PRP, which suggests that in some patients there is an overlap between these two diseases.^7^

Regarding dermoscopy, the features described include a pink background with polymorphous vessels (glomerular, dotted, lineal, and hairpin) and star- or oval-shaped yellow-white structures with a white halo, besides scaling.^8^, ^9^

Typically, histopathology shows focal acantholysis and different degrees of dyskeratosis. There are four histologic subtypes: Darier’s disease-like, pemphigus-like, Hailey-Hailey-like, and spongiotic. These subtypes can appear alone or coexist.^1^, ^10^

First-line treatment consists of the use of emollients and topical steroids and vitamin D analogs, associated with H1 antihistamines. Therapy with systemic retinoids, oral steroids, or phototherapy is reserved for extensive or treatment-resistant cases. In the present case, narrowband UVB phototherapy was used, with a successfully response after 18 sessions.

## Financial support

None declared.

## Authors’ contributions

Pablo Vargas-Mora: Approval of the final version of the manuscript; conception and planning of the study; elaboration and writing of the manuscript; obtaining, analyzing, and interpreting the data; effective participation in research orientation; critical review of the literature; critical review of the manuscript.

Diego Orlandi: Approval of the final version of the manuscript; conception and planning of the study; elaboration and writing of the manuscript; obtaining, analyzing, and interpreting the data; effective participation in research orientation; critical review of the literature; critical review of the manuscript.

Irene Araya: Approval of the final version of the manuscript; conception and planning of the study; elaboration and writing of the manuscript; obtaining, analyzing, and interpreting the data; effective participation in research orientation; critical review of the manuscript.

Claudia Morales: Approval of the final version of the manuscript; conception and planning of the study; obtaining, analyzing, and interpreting the data; effective participation in research orientation; critical review of the manuscript.

## Conflicts of interest

None declared.
